# Exploring the experience of managers, employees, and pharmacists in clinical pharmacy in primary care using the SEIPS model: A focus group study

**DOI:** 10.1016/j.rcsop.2025.100657

**Published:** 2025-09-14

**Authors:** Karin Svensberg, Lea Axelsson, Lina Hellström

**Affiliations:** a Department of Pharmacy, Uppsala University, Uppsala, Sweden; b Hospital Pharmacy Department, Uppsala University Hospital, Uppsala, Sweden; c Pharmaceutical Department, Region Kalmar County, Sweden; d Department of Medicine and Optometry, Faculty of Health and Life Sciences, Linnaeus University, Kalmar, Sweden

**Keywords:** Pharmacists, Primary health care, SEIPS-model, Quality improvements, Interprofessional teams, Focus groups

## Abstract

**Background and aim:**

Medication management in primary care faces challenges that affect patient outcomes.

The inclusion of clinical pharmacists in care teams aims to address these issues. In Nordic countries, the role of clinical pharmacy services in primary care is still evolving with limited research. The Systems Engineering Initiative for Patient Safety (SEIPS) model provides a framework for evaluating healthcare systems by examining the system factors and processes that influence outcomes. This study aimed to identify factors influencing the integration and advancement of the role of pharmacists in primary care using the SEIPS model.

**Methods:**

A focus group study was conducted with four groups (*n* = 17): managers, pharmacists, nurses, and general practitioners. Deductive thematic analysis guided by the SEIPS model was used to structure the data.

**Results:**

Despite taking time to establish the pharmacist as a colleague, the participants expressed satisfaction with the collaboration and the pharmacist's role within the work system and processes at healthcare centres. Central factors for system and process development were identified, including a needs-based and structured approach to implementation, pharmacists with the right qualities and skills, teamwork, and physical presence. Perceived outcomes included increased pharmaceutical knowledge among coworkers and patients, reduced workload for staff, and improved patient safety regarding therapies.

**Conclusion:**

Participants emphasised the potential of integrating pharmacists into primary care to address medication-management challenges. The SEIPS model provides insights into work system dynamics and can help develop the role of pharmacists in healthcare.

## 1 Introduction

Medication management in primary care presents several challenges, including an increasing number of elderly and frail patients with complex treatment regimens, care transitions, frequent medicine shortages, and escalating medication costs.^1^, ^2^, ^3^ These factors may increase the risk of adverse drug events, and increase the workload related to medication management. At the same time healthcare workforce shortages are a problem.^4^ In Sweden, regions and municipalities share responsibilities for primary care and there are both public and private providers. Municipalities finance and provide basic nursing healthcare for patients who receive social services or for elderly individuals in their own or special housing. The regions finance and govern primary care for the general population in primary care centres (PCC), also called general practice. PCCs are administered by a team of healthcare professionals such as general practitioners, nurses, and physiotherapists.^5^ The inclusion of clinical pharmacists as an integral part of such PCCs teams has been suggested as an important step in developing practices and managing challenges with the aforementioned medication management issues.^6^ In addition, pharmacists are viewed as underutilised professional resources. This has led to an initial evolution of the role of pharmacists in primary care in certain countries, such as the United States, the United Kingdom, Australia,^4^ and more recently, Nordic countries.^7^, ^8^, ^9^, ^10^, ^11^, ^12^ Training pathways for clinical pharmacy careers vary worldwide.^13^ In Sweden, an undergraduate five-year master's degree in pharmacy provides pharmacists direct access to perform clinical activities. Within that undergraduate education optional clinical pharmacy courses are offered. In addition, one university offers a one-year postgraduate master's program in clinical pharmacy. Patient-centered clinical pharmacy services have become well established in Swedish hospitals over the last 10 to 15 years.^14^ Admission medication reconciliation and medication reviews are common hospital-based tasks. In contrast, the concept of pharmacists working in general practice is relatively new.^9^^,^^14^ The implementation and funding of clinical pharmacy services are decided by the 21 regional health authorities (“regions”), leading to variation across the country. The number of clinical pharmacists in Swedish primary care remains limited, and positions are most often part-time.^14^

In countries that have well-established pharmacists working in PCCs, they undertake patient-centred activities, most often medication reviews, and activities at the provider level, such as education and support for rational medication use.^4^^,^^15^ Evidence suggests that integrating pharmacists into primary care may have a positive impact on quality of prescribing and may also reduce general practitioner workload while the impact on hospital attendance or admissions is uncertain^16^, ^17^, ^18^; according to a recent rapid review^4^ no studies described any negative outcomes. Interview studies from outside Nordic countries have found that physicians view the integration of pharmacists into primary care as mostly positive.^19^, ^20^, ^21^, ^22^ They value pharmacists' medication-related expertise and perceive benefits such as improved patient outcomes, access to care, reduced provider burden and cost savings. Other stakeholders, such as nurses, receptionists, managers, and patients also have positive experiences.^23^^,^^24^ The challenges that pose barriers are attitudinal, professional, and logistical.^24^ Knowledge of the views and experiences of those working with clinical pharmacists can contribute to further develop collaboration models. In Nordic countries, clinical pharmacy services in primary care are still in the developmental stage; research is scarce, and there is a need to continue studying the pharmacist's role therein.

**Fig. 1:**
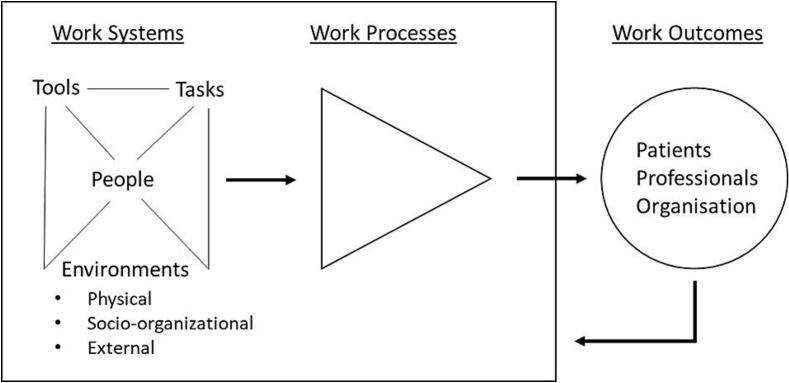
The relationship between the three components of the SEIPS model: work systems, work processes, and outcomes. The figure also depicts the subcomponents of the work systems: tools, tasks, people, and environment. Outcomes affect patients/families, the professionals or the organisation. The figure is self-drawn based on SEIPS 101 (25) (Created using Microsoft Powerpoint).

The Systems Engineering Initiative for Patient Safety (SEIPS) is a model that offers a structured framework to evaluate and develop a healthcare system^25^ by comprehensively describing the elements within the system that influence outcomes. SEIPS has been previously applied in healthcare research, including in medication management.^26^ Several versions of the SEIPS exist with slightly different focuses,^25^^,^^27^, ^28^, ^29^ but all versions are divided into three components: work systems, work processes, and work outcomes. Fig. 1 illustrates the relationship between these components. The work system in SEIPS is described as *“an individual performs a series of tasks using various tools and techniques. The execution of these tasks occurs within a specific physical environment under specific organizational conditions*”.^25^ The work process describes how the work operates based on the structure of the work system. Work processes are physical, cognitive, social-behavioural, or a combination of these. The process and work system are intertwined and cannot be separated; the process is embedded in the work system.^27^ Together, the work system and work process lead to the work outcome focusing on the impact on the patient, staff, and organisation. A PCC is a work system in which a clinical pharmacist is involved in different work processes, together with other health care professionals (HCP), such as medication reviews and prescription renewals, resulting in different work outcomes. The overall aim of implementing clinical pharmacy services in PCCs is to improve medication-related processes and outcomes by enabling the performance of specific tasks using appropriate tools. Therefore, we aimed to elaborate on the experiences of clinical pharmacy services among primary care staff in Swedish PCCs using the SEIPS model.

## 2 Aim

This study aimed to identify factors influencing the integration and advancement of pharmacists' roles in Swedish primary care centres, using the SEIPS model to explore the work system, processes and outcomes.

## 3 Method

### 3.1 Study design and context

This study is part of a quality improvement evaluation of clinical pharmacists in primary care in the Kalmar County Region. Focus groups were selected as the data collection method, as this is an appropriate method for exploring experiences, allowing for participants to interact and discuss different viewpoints.^30^ Additionally, focus group discussions are useful when the final outcomes can affect the participants themselves, which applies to this study. The SEIPS101 model,^28^ a practice-oriented version of SEIPS, was chosen as analytic framework. This study was conducted according to the COREQ checklist.^31^ Other potential frameworks are Donabedian's model or the Consolidated Framework for Implementation Research (CFIR).^32^^,^^33^ SEIPS builds on Donabedian's model but adds an explicit focus on the work system, making it more suitable for studying the integration of pharmacists in primary care. CFIR is valuable for identifying barriers and enablers in implementation, but as our focus was on work processes rather than broader contextual determinants, SEIPS was deemed more appropriate.

Since 2021, four pharmacists employed by the Pharmaceutical Department of the Region Kalmar County have worked part-time at four public practices, each at 50 % capacity. Their positions were funded by the primary healthcare sector but were structured as project-based roles, with no guarantee of permanent funding. All clinical pharmacists had a postgraduate degree in clinical pharmacy and 6–15 years of clinical experience. The PCCs had between 25 and 35 employees, mainly physicians, nurses, assistant nurses, and medical secretaries, and served approximately 7000–8500 patients each. The pharmacists became involved in a wide range of activities, including direct patient consultations, collaborative medication reviews with physicians, staff education, and participation in quality improvement initiatives. A more detailed descriptions of their roles are presented in Table 2 as part of the results.

### 3.2 Study population and recruitment

The study population consisted of managers, physicians, nurses, and clinical pharmacists from four PCCs with an active clinical pharmacist. Potential focus group participants received written information about the study via email or in person. All four managers and all four pharmacists were asked to participate and agreed to it. We informed all general practitioners and nurses at the PCCs about the study. They were eligible to participate if they collaborated actively with a clinical pharmacist. The selection of the number of employee participants was guided by practical and methodological considerations. Due to time constraints, each focus group session was limited to 60 min, which restricted the number of participants to ensure that everyone had sufficient opportunity to contribute. Additionally, only a subset of physicians and nurses could be released from clinical duties to participate. Given that the research question was relatively focused and not highly complex,^45^ conducting two focus groups was considered sufficient to capture diverse perspectives. We therefore aimed for groups of 6 participants, which we deemed optimal for facilitating in-depth discussion within the allotted time.^30^ Eight general practitioners and six nurses showed interest in participating; among them, 12 participants were selected (purposive sampling). Employee focus groups were created by combining two PCCs. Shortly before the focus groups were conducted, three participants reported being unable to participate. In the final sample of nine employees, there was at least one physician and one nurse in each PCC. The final four focus groups were conducted in October 2022 as follows: focus group 1: four managers (three females, one male); focus group 2: four pharmacists (four females, zero males); focus group 3: four participants, including two general practitioners and two nurses (two females and two males); and focus group 4: five participants, including three general practitioners and two nurses (four females and one male).

### 3.3 Data collection

The interview guide was based on the SEIPS components.^28^ A core question and supporting questions were developed for each section (Table 1, see Appendix 1 for the full interview guides and interview mind maps). The guide included a warm-up question, main interview section, closing question, and closing information. The authors discussed the interview guide to ensure that it effectively captured the desired information for the study. Because of time constraints, a pilot interview could not be undertaken. Data were collected during the focus groups by a moderator (LA) and co-moderator. At the conclusion of each focus group, the co-moderator provided a summary to allow participants the opportunity to agree, disagree, or emphasise the points they deemed important. The focus groups lasted between 46 and 72 min, and the discussions were audio-recorded. The focus group with managers took place as a video meeting via Microsoft® Teams (version 1.5.00.33362, 64-bit), while the remaining groups were held in person at Kalmar County Hospital or one of the PCCs.

**Table 1:** Simplified core questions for each component and subcomponent.

Component and subcomponent		Core question (example)
Work system	*Person*	• How does/doesn't the pharmacist/you as a pharmacist contribute with pharmaceutical expertise to the PCC?
	*Environment*	• How clear is the pharmacist's role at the PCC?
	*Task*	• Describe the tasks and responsibilities of the pharmacist at the PCC.
	*Tools*	• Have any obstacles emerged for the pharmacist in carrying out their duties, such as access to different systems?
Work process		• How has the collaboration between the pharmacist and colleagues at the health centre been? Describe a typical day.
Work outcomes		• What is your perception of how the pharmacist has contributed to patient care?

PCC = primary care centres.

### 3.4 Reflexivity

The last author, LH (PhD and clinical pharmacist), was actively involved in project development and implementation and consequently had a professional relationship with several of the participants. This contributed to a deeper understanding of the participants' reasoning but also entailed preconceptions that may influence the analysis and interpretation of data. Throughout the research process, LH critically reflected on her preconceived assumptions to remain as neutral as possible. KS (PhD), a pharmacist, had not been involved in the project or worked in primary care; however, KS conductes research on the expanding roles of pharmacists. The interviewer (LA), a pharmacy master's student, had prior clinical placement experience where pharmacists were positively received. She was aware of potential scepticism from other professionals and reflected on her influence during interviews. LA conducted the interviews as part of her thesis. Throughout the process, she engaged in reflexive consideration of her potential influence, on e.g. data collection, by emphasizing her role as a student and welcoming both positive and negative responses.

### 3.5 Data analysis

A deductive thematic analysis inspired by the “Framework Method”, and based on the SEIPS theoretical model, was employed.^34^ The components and subcomponents of the model served as the analytical framework. However, there was room to discover unexpected aspects that could become themes beyond the predetermined ones. In Step 1, audio recordings were transcribed. In Step 2, the authors familiarised themselves with the content by reading the transcripts multiple times. Notes taken by the co-moderators during the focus groups were reviewed and reflected. Steps 3–5 involved creating a codebook by describing the components and subcomponents of the SEIPS model. Taguette (version 1.3.0) was used to code textual data from the transcripts according to the codebook. Coding was performed by LA and discussed with KS and LH to ensure consistency. After all transcripts were coded, the text data were charted in Step 6 using Microsoft® Excel (Microsoft 365 MSO, version 2211) and sorted according to SEIPS. In Step 7, the data were interpreted by all authors, and the transcripts were re-read. Additional inductive categories were identified for the outcome components. The work system and process components were collapsed as they significantly influenced each other. The data were presented in the form of analytical texts and quotes.

### 3.6 Ethical considerations

No ethical approval from the Swedish Ethical Review Authority was required, as the study did not involve sensitive personal data.^35^ This study was approved by the primary care director and the pharmaceutical manager of the Pharmaceutical Department of Region Kalmar County. In addition, it was conducted according to the General Data Protection Regulation because personal data, such as contact information and workplace details, were collected. The participants were given clear information about the study and their consent was obtained. Participants were also informed that they were not to repeat what was said in the focus groups. Data were handled confidentially and stored securely.

## 4 Results

The results are presented according to the three components of SEIPS: work system, work processes, and outcomes, as no additional components were identified. Quote abbreviations include focus group (FG) with a number indicating the specific group, and manager (M), pharmacist (Ph), general practitioner (GP), or nurse (N) with a number specifying the individual in the group.

### 4.1 Work system and work processes

#### 4.1.1 Person

Employees and managers expressed appreciation for the pharmacists' expertise, believing that the unique contributions of pharmacists could not be matched by other professions in the system. One physician noted:”*I can prescribe, and I am familiar with many medications at an overview level. But she has a deeper pharmaceutical knowledge, and she should have that, more than I do. So, I think she definitely adds knowledge to the knowledge bank*.” (FG4; GP3).

A similar view was expressed by one of the managers: “*In special housing [conducting medication reviews] this profession adds another dimension and more depth.*” (FG1; M1)*.* Still, pharmacists emphasised that the need for competence development should be evaluated before undertaking certain assignments, given their varied work experiences. Both employees and pharmacists stressed the importance of prior clinical work experience in hospitals, including interdisciplinary collaboration. Expertise in specific therapeutic areas such as geriatrics and cardiology were highly valued.

#### 4.1.2 Tasks

The tasks in the SEIPS model are specific actions within larger work processes,^26^ see Table 2. Choosing which tasks the pharmacists should perform were initially challenging. Prior to project start, the involved pharmacists, their coordinator and manager in the Pharmaceutical Department prepared a list of tasks to be performed. Despite this, some employees experienced a high degree of unclarity regarding possible tasks for pharmacists and suggested clearer suggestions for tasks to choose from when pharmacists were implemented at a new PCC (in a new system). The pharmacists, and to some degree, the managers, expressed that they were familiar with possible tasks but had experienced difficulties in clearly defining the needs of the PCC that pharmacists could meet.

**Table 2:** Examples of processes and tasks performed by the pharmacists at the primary care centres (PCC). Based on information that emerged from the focus groups.

Overarching process	Tasks performed by pharmacists
Medication review for patients	- *Elicit information from the EHR*
• in special housing	- *Elicit information from other HCPs*
• living at home with home health care	- *Meet and counsel the patient*
	- *Analyse the medication treatment*
• living at home independent of help from others	
	- *Communicate with the physician*
	- *Document in the EHR*
	- *Follow-up with other HCPs and/or the patient*
Prescription renewals	- *Elicit information from the EHR*
	- *Counsel the patient*
	- *Communicate with the physician*
General medication management	- *Being a resource in daily medication issues*
	- *Writing routines (*e.g. *for treatment with osteoporosis drugs, or for prescription renewals)*
Education and training for patients and employees	- *Writing patient information material*
	- *Performing patient education (diabetes)*
	- *Inform and update colleagues at the PCC*
Quality improvement work	- *Withdraw statistics on prescribing*
	- *Evaluate certain processes, such as medication reviews*

EHR = electronic health record, HCP = healthcare professional.

A pharmacist described it as “*...not entirely easy to communicate in which cases I can contribute, since it [clinical pharmacist] is a new profession. So, I think that has taken some time.*” (FG2; Ph4)*.*

Reasons why it was challenging to decide on work tasks included that other staff were not aware of pharmacists' competence and working methods, and that pharmacists previously mostly worked in hospitals (see also Environments).

“*Yes, it takes a long time settling into the tasks. It's something completely different from working in a hospital*.” (FG2; Ph3). On the other hand, it was also regarded as positive that implementation had taken some time: “*I think it's also good when you can feel your way...//*” (FG3; GP1)*.* In discussions about future tasks, the focus groups suggested that pharmacists could play a more marked role in the process of deprescribing drugs such as addictive drugs and be responsible for medication-related training courses and skill development for primary care colleagues.

#### 4.1.3 Tools

As pharmacists are not always present in PCC, employees often use digital communication tools to ask questions. A drawback of digital communication was that pharmacists were always available, which increased their workload and was sometimes perceived as stressful.

#### 4.1.4 Socio-organizational environment

The participants thought that the implementation process may have been hindered by the fact that the assignment occurred part-time, contributing to a sense of fragmentation and slow progress. However, a full-time position, was not considered viable. To overcome some difficulties associated with the introduction of a new profession, the managers recommended the development of an introductory program akin to those used in other professions. The pharmacist group stressed the need to raise awareness of their clinical skills among other professionals.

Team based working processes was seen as an important factor in establishing the role of pharmacists. In one PCC, medication reviews were not based on teamwork; instead, the pharmacist met the patient and sent a message to the physician. This was time-effective, but both physicians and pharmacists emphasised that verbal communication and discussion are often better for complex patients, and they expressed a wish to strengthen multidisciplinary collaboration in their PCC. A pharmacist said: “...*for me it would have worked better or been smoother if I had been part of the team from the beginning*” (FG2; Ph3)*.*

Other facilitating factors for successful collaboration and establishment of personal relationships with each other that was reported, were physical presence, enabling work atmosphere at the PCC and trust in individual pharmacists. One nurse said, “…*and having a person on-site that you can turn to for help, for example, with medicine shortages or any other medication-related question or so. Someone you know is there and you can go knock on their door and ask*.” (FG3; N1).

In general, pharmacists felt respected in their new workplace and the overall climate was considered permissive. It emerged, however, that pharmacists are sometimes perceived as external individuals from pharmacies rather than colleagues. Regarding the shift in role and responsibility for certain tasks from doctors or nurses to pharmacists, employees said they had full confidence in the pharmacists and were hence comfortable delegating responsibilities to the pharmacist, such as follow-ups on medication adjustments, even if this had not yet become a reality at all PCCs. This is an example of how changes in the s*ocio-organizational environment* led to changes in work processes. Managers organised their collaborations with pharmacists in slightly different ways. Units that had regular meetings between the manager and pharmacist believed that they facilitated the smooth development of the pharmacist's tasks and involvement in various processes.

#### 4.1.5 External environment

In the groups with managers and employees there were discussions about how and by whom decisions about pharmacist tasks should be made when introducing or developing clinical pharmacy services. They considered it important to make use of previous experience, and they also considered it important to start with the work already conducted by the Regional Drug and Therapeutics Committee to improve the quality of prescriptions. They also suggested that the exchange of experiences among PCCs could be facilitated through joint meetings. However, they preferred local decisions at the PCC to prioritize tasks and stressed the importance of a tailored and needs-based approach.

One manager said, “*I believe that different PCCs have different needs. // So, I would wish that there could be some freedom in that. // And that you sit down together with the doctor group and the nurse group, those who are relevant, and me, plus the pharmacist, and set goals together*.” (FG1: M3).

Pharmacists highlighted the advantages of being situated at a single PCC over operating across multiple sites. Simultaneously, they recognised the potential benefits of enhanced collaboration across sites, suggesting collective referral management by a group of pharmacists with diverse expertise.

### 4.2 Work outcomes

#### 4.2.1 Enhanced medication knowledge

In all focus groups, it was evident that the presence of a pharmacist in the work system affected the level of medication knowledge at the PCC. Employees mentioned that they learned a lot through direct communication with pharmacists and benefited from the information they provided. One physician stated: “*Yes, I see that it also leads to competence development within the group.*” (FG3; GP2). Managers and employees said that many patients expressed a desire to discuss their medications. Having an onsite pharmacist was considered an effective way to address this need, especially given the existing workload of doctors and nurses. One manager said: “*For me, it has primarily been that I feel the patient has really come into focus, and that she has been such a great asset in providing patients with knowledge and understanding about their medications.*” (FG1; M3).

#### 4.2.2 Increased medication awareness

Both employee and pharmacist focus groups thought pharmacists heightened overall medication awareness among PCC staff. In one employee group, this was described as a domino effect, with the presence of a pharmacist leading to an increased focus on medication. This, in turn, prompted more deliberate choices by the staff, contributing to enhanced treatment quality. A nurse and physician discussed this as follows:

N1: “*A domino effect, so to speak. You check the medication list when you have the patient on the phone ... // It creates an awareness around it.*”

GP2*:* “*The focus is increasingly on medication when we have a pharmacist on-site, I think. It becomes a natural part of it.*” (FG3).

Pharmacists mentioned that after working at the PCC for a while, they felt that awareness of the medication aspect among the staff was always present without needing to say anything.

#### 4.2.3 Reduced workload

According to employees, the pharmacist provided valuable support in reviewing medication treatments. Across all groups, it was evident that physicians at the PCC were overburdened and lacked the time to address all medication-related needs. The pharmacist played a crucial role in alleviating the employee workload by providing both time and task support. One focus group physician mentioned the following:

“*Because we don't always have enough time. // …but then you can pass it on and get help with all sorts of things related to this.*” (FG4; GP1)

In another focus group, a physician and a nurse agreed: GP2: “*[...] So I don't mind letting go of certain tasks. I just see it as an advantage since it provides some relief.*” *N1:* “*Yes, exactly.*” (FG3).

#### 4.2.4 Improved patient safety

All focus groups emphasised that having a pharmacist at the PCC enhanced patient safety, for example by reducing the risk of medication errors. This improvement resulted from activities, such as reviewing medication lists and conducting patient consultations.

“*…there's always a risk that if the patient is hospitalized, the medication list is just copied, and the patient is given medications according to that list. So, there's a patient safety aspect to all of this. If you've done a medication review and the list has been updated, it increases safety.*”

*(FG3; N2)*

Pharmacists noted that new and sometimes crucial information about medication treatment often surfaced during consultations, and that information was not always revealed during doctor visits. For example, during home visits, additional information about the practical handling and actual use of medicines, including over-the-counter medicines, often emerged, and this added value for certain patients. Additionally, pharmacists believed that they could proactively address preventive measures in primary care, encountering patients earlier, and in more stable life phases than in hospital care. In addition, the managers reported that medication-related work at the PCC became more structured and prepared in the presence of a pharmacist.

## 5 Discussion

As healthcare systems increasingly seek to integrate pharmacists into primary care, understanding the conditions that support successful implementation becomes essential. This study illustrates how system-level factors such as social and organizational dynamics, physical presence, and task design influence the integration of pharmacists into primary care teams. Although previous studies have described the tasks performed by pharmacists in primary care in Sweden,^9^^,^^11^^,^^36^ our study extends this by placing these tasks within a system and process context. Including multiple professional perspectives revealed both enabling and limiting factors that affect integration. The discussion below focuses on the facilitating factors identified in the study and how these factors can reinforce the components of the SEIPS model, ultimately contributing to strengthening the pharmacist's role in primary care.

### 5.1 A needs-based and structured approach to implementation

Our findings show that unclear expectations and limited communication about pharmacists' roles contributed to a slow start in the implementation process. While this delay was at times seen as beneficial for adaptation, it also caused uncertainty among team members. These insights support a needs-based implementation approach, with flexible adaptation to local contexts. Similar conclusions have been drawn in Dutch and UK studies, where early role clarity and stakeholder engagement were key to successful implementation,^37^^,^^38^ one must allow the role of the pharmacist to be tailored to meet the unique requirements of each general practice and acknowledge that the integration process requires time.^23^^,^^38^ From a SEIPS perspective, this reflects the interaction between organizational factors (e.g., leadership communication), tasks (e.g., defined responsibilities), and the social environment (e.g., team acceptance). When these elements are not aligned, the work system hinders rather than supports integration. An aspect not discussed during the focus groups but previously shown by Claire et al.^39^ is that general practitioners or senior clinical pharmacists, as clinical mentors, are vital to the success of the clinical pharmacists' role. Another success factor was a nominated person who supported the pharmacist and role implementation, especially during the early pilot phase. These measures, which are mainly associated with environment and task components, contribute to a more structured implementation.

### 5.2 A pharmacist with the right qualities and skills for fostering trust

A recurring theme in our findings was the importance of trust in enabling pharmacists to take on a meaningful role within the primary care team. Participants described that trust developed gradually and was influenced by the pharmacist's personal qualities, clinical competence, and ability to communicate effectively. Hurley et al. 2022^40^ suggested that a combination of prior hospital and community experiences is particularly desirable. In addition, consultations and clinical reasoning skills are essential.^37^ This highlights a critical dynamic within the SEIPS work system: the interplay between the person (skills, knowledge, experience), the social/organizational environment (colleague relationships, expectations), and task allocation. When these components align, such as when pharmacists demonstrate confidence and competence in patient-related activities, trust is built, and collaboration becomes more fluid. Previous work experience and personal qualities are also important for the pharmacists' comfort in their roles.^24^ Only when the pharmacists feel confident in their role and colleagues trust them can responsibilities be delegated to the pharmacist, allowing them to develop their position and engage in new work processes. As clinical competence was identified as one central factor, less-experienced pharmacists might be less prepared to tackle their roles. Thus, a structured training program is essential to facilitate effective integration into the work system and enable them to fulfil their roles.^23^^,^^37^^,^^40^^,^^41^

### 5.3 Teamwork and physical presence as facilitators for mutual understanding of roles

In the result, team-based work and physical presence were key enablers for building a mutual understanding of roles between pharmacists and other healthcare professionals. In particular, shared tasks for example medication reviews were highlighted as opportunities where interprofessional collaboration became both necessary and productive. When pharmacists were physically present at the PCC, interaction increased naturally, which in turn fostered role clarity and smoother collaboration. These findings are supported elsewhere.^22^^,^^42^ Similarly, role clarity coincides with the concept of identity alignment which, according to a recent study,^38^ is essential for the success of a new interprofessional healthcare model involving clinical pharmacists in general practice. Identity alignment is enhanced by working within the same team and sharing the same physical space.

Although physical presence appears to be important for integration,^7^^,^^9^^,^^40^ resource constraints are a challenge that also needs to be addressed when expanding clinical pharmacy services, and remote work may allow for a better allocation of resources and access to more patients.^43^^,^^44^ Further research and development of clinical telepharmacy initiatives as alternative models for delivering care are required. The development should be guided by the principle of ‘digital when possible, physical when necessary’, and mutual understanding of roles might be even more important to achieve.

## 6 Methodological discussion

This study is subject to some limitations. One risk is that participants may have felt compelled to participate in the study because of the small number of eligible participants. Additionally, this study only incorporated the perspective of the HCP and not the patient, and future studies should include the patient perspective to capture all nuances when implementing a new role. The structure provided by the SEIPS model can contribute to transparency in the analytical process and an understanding of the connections between raw data and final results in different categories, which can enhance reliability.^34^ The SEIPS framework is complex and often applied with input from human factors expertise. As our research group did not include such expertise, we applied the simplified SEIPS 101 model,^28^ which facilitated feasibility and provided an accessible tool to describe work system components and interactions. While this approach may have limited the analytical depth and introduced bias in how the model was operationalized, the inclusion of expertise in the local primary care context strengthened the contextual validity of the analysis. Nevertheless, the descriptive nature of SEIPS, and its lack of explicit guidance on which elements to prioritize,^28^ places considerable reliance on user interpretation and may constrain the transferability of findings. The choice of profession-specific focus groups aimed to avoid power imbalances. For example, pharmacists were interviewed separately as they were partly being evaluated, and managers were also kept in separate groups to avoid hierarchical pressure.

Considering the concept of information power,^45^ our use of a predefined model reduces the number of participants needed, complemented by a focused aim and case study design. In this case, the moderator lacked experience, which may have required additional focus groups to gather sufficient data. This was not possible. Instead, a co-moderator (a pharmacist and researcher with experience in conducting focus groups) was present to provide support. After the focus groups, the moderator and co-moderator briefly discussed their thoughts on how they had gone and summarised them. We believe that the sample specificity was strong, as all participants had experience working with a pharmacist and all pre-defined professions were represented. The two focus groups with employees yielded similar overall findings, suggesting a degree of meaning saturation.^46^ However, it is possible that participants who were more positively inclined towards collaboration with a pharmacist were overrepresented. We did not undertake renewed recruitment among those who had declined participation, nor did we explore their reasons for non-participation. Such perspectives might have contributed additional insights. All eligible managers and pharmacists were included.

Further, several individuals were involved in data analysis, which added reflexivity to it. Additionally, two participating pharmacists reviewed the results section, contributing to member checking. Therefore, we argue that the information power and “saturation” of our study is reasonable for addressing its aim.

### 6.1 Future perspectives

The clinical pharmacists in this study adopted a broad and flexible role. As a result, they became involved in a wide range of activities. This development highlights the adaptability of clinical pharmacists and their potential to contribute to multiple aspects of patient care and healthcare system development in primary care. Internationally, however, clinical pharmacy models vary considerably. In some, the pharmacists have a more limited role, for example conducting medication reviews in a certain group of patients.^4^ An integrated care model similar to ours has been implemented in the Netherlands, although their pharmacists had a full-time employment enabling an even broader role and responsibility.^37^ These differences underscore that clinical pharmacy models differ not only in scope of activities but also in level of integration, funding structures, and team collaboration. More research is needed to evaluate which practice models are most beneficial for patients and practices, and under what circumstances broad or more focused roles are most effective.

Applying frameworks such as SEIPS already in the planning phase can not only help analyse existing processes but also guide the design of new models, by systematically mapping how pharmacists' activities interact with other professionals, workflows, and patient needs. To understand care processes, we need to go beyond describing tasks to assess the entire work system.^27^ This was evident from the present study, as among the subcomponents of the work system, the socio-organizational environment demanded high attention to ensure the successful integration of pharmacists in primary care. Hence, it is important to consider the factors identified during both the planning phase and active implementation of introducing a pharmacist into a PCC or similar healthcare system with or without utilising the SEIPS model. Using the SEIPS model during the planning phase could allow for systematic mapping of potential work processes, enabling a better understanding of where pharmacists' skills can be optimally employed. Moreover, by hypothesising potential outcomes during the planning phase, it is possible to reverse engineer how the pharmacist works most effectively to achieve these goals, in collaboration with other team members. Thus, it can be used to identify key processes that inform best practices of pharmacists in these settings. For example, if a hypothesised outcome is to improve medication safety, one can plan backwards by structuring workflows that ensure pharmacists systematically review high-risk medications at defined points in the care process. Similarly, if fostering interprofessional learning is a goal, planning could include joint debrief sessions after pharmacists' activities, creating structured opportunities for staff to reflect and learn from the pharmacist's input. In this way, SEIPS can be used not only to identify key processes but also to design them in alignment with intended outcomes, thereby informing best practices for pharmacists in these settings. While one could argue that any intervention of a new service can (and should) be designed backwards with its goals in mind even without a formal model, SEIPS could here offer a structured systems perspective that helps to make explicit how different work system elements and processes interact. This can strengthen the focus on workflows and causal relationships—clarifying what affects what—which may otherwise risk remaining implicit. At the same time, SEIPS should not be seen as a universal solution; if time and resources allow, it can be complemented with other frameworks to capture additional dimensions and thereby ensure maximal effect of the new model/service. Nevertheless, we propose that the SEIPS model can be employed to evaluate the performance and integration of clinical pharmacists within long-established systems, ensuring that their contributions are optimised and aligned with the organizational goals of a specific PCC or a similar setting. As a next step in the PCCs in our study, there is a need to identify problems or patterns to address to optimize the process of medication reviews. This could be done using a journey map, one of the SEIPS tools intended to be used also by practitioners.

## 7 Conclusion

Pharmacist integration can be facilitated through thorough pre-implementation planning of potential tasks, clear communication of these tasks, a structured introductory program, and a need-based approach to implementation. To achieve trust in pharmacists, managers should ensure that the appropriate individual with deep pharmaceutical knowledge and clinical experience is integrated into the system. Additionally, the physical presence of pharmacists and team-based work environments are crucial for establishing and advancing their roles in primary care. These findings may help inform policy decisions and support the development of tailored strategies to further expand the role of clinical pharmacists within primary healthcare systems.

## CRediT authorship contribution statement

**Karin Svensberg:** Writing – original draft, Visualization, Supervision, Resources, Methodology, Investigation, Formal analysis, Data curation. **Lea Axelsson:** Writing – review & editing, Investigation, Formal analysis. **Lina Hellström:** Writing – original draft, Supervision, Project administration, Methodology, Investigation, Formal analysis, Data curation, Conceptualization.

## Disclosure statement

The authors report there are no competing interests to declare. This research did not receive any specific grant from funding agencies in the public, commercial, or not-for-profit sectors.

## Declaration of generative AI and AI-assisted technologies in the writing process

During the preparation of this work the authors used ChatGPT 3.5 to improve language accuracy. After using this tool/service, the authors reviewed and edited the content as needed and take full responsibility for the content of the publication.

## Declaration of competing interest

The authors declare that they have no known competing financial interests or personal relationships that could have appeared to influence the work reported in this paper.

## Data Availability

The data used and analysed in the current study are not available owing to ethical restrictions.

## Appendix A Supplementary data

Interview guides and interview mind maps. Image 1

## References

[1] Kallio S., Kumpusalo-Vauhkonen A., Järvensivu T., Mäntylä A., Pohjanoksa-Mäntylä M., Airaksinen M. (2016). Towards interprofessional networking in medication management of the aged: current challenges and potential solutions in Finland. Scand J Prim Health Care.

[2] Lee C.Y., Goeman D., Beanland C., Elliott R.A. (2019). Challenges and barriers associated with medication management for home nursing clients in Australia: a qualitative study combining the perspectives of community nurses, community pharmacists and GPs. Fam Pract.

[3] Liew T.M., Lee C.S., Goh S.K.L., Chang Z.Y. (2020). The prevalence and impact of potentially inappropriate prescribing among older persons in primary care settings: multilevel meta-analysis. Age Ageing.

[4] Karampatakis G.D., Patel N., Stretch G., Ryan K. (2024). Integration and impact of pharmacists in general practice internationally: a rapid review. J Health Serv Res Policy.

[5] The National Board of Health and Welfare (2020). About the Swedish healthcare system. https://www.socialstyrelsen.se/en/about-us/healthcare-for-visitors-to-sweden/about-the-swedish-healthcare-system/.

[6] NHS England (2016). General practice forward view. https://www.england.nhs.uk/wp-content/uploads/2016/04/gpfv.pdf.

[7] Blondal A.B., Sporrong S.K., Almarsdottir A.B. (2017). Introducing pharmaceutical care to primary Care in Iceland-an Action Research Study. Pharmacy.

[8] Holst S.S., Hansen J.M., Kaae S., Vermehren C. (2024). Development of a medication review intervention by seconding a hospital pharmacist to primary care. Explor Res Clin Soc Pharm..

[9] Kempen T.G.H., Koumi R., Sporrong S.K. (2023). Pharmacists in general practice: what do they do? A qualitative case study. Int J Clin Pharm.

[10] Lenander C., Elfsson B., Danielsson B., Midlöv P., Hasselström J. (2014). Effects of a pharmacist-led structured medication review in primary care on drug-related problems and hospital admission rates: a randomized controlled trial. Scand J Prim Health Care.

[11] Wickman K., Dobszai A., Modig S., Bolmsjö B.B., Caleres G., Lenander C. (2022). Pharmacist-led medication reviews in primary healthcare for adult community-dwelling patients - a descriptive study charting a new target group. BMC Prim Care.

[12] Bø K.E., Halvorsen K.H., Risør T., Lehnbom E.C. (2023). Illuminating determinants of implementation of non-dispensing pharmacist services in home care: a qualitative interview study. Scand J Prim Health Care.

[13] Moura L., Costa A., Steurbaut S., Mota Filipe H., Leite S., Alves da Costa F. (2003). Exploring worldwide training pathways that enable clinical pharmacy career development. J Am Pharm Assoc.

[14] Eriksson T., Melander A.C. (2021). Clinical pharmacists’ services, role and acceptance: a national Swedish survey. EurJ Hosp Pharm.

[15] Hazen A.C.M., de Bont A.A., Leendertse A.J. (2019). How clinical integration of pharmacists in general practice has impact on medication therapy management: a theory-oriented evaluation. Int J Integr Care.

[16] Croke A., Cardwell K., Clyne B., Moriarty F., McCullagh L., Smith S.M. (2023). The effectiveness and cost of integrating pharmacists within general practice to optimize prescribing and health outcomes in primary care patients with polypharmacy: a systematic review. BMC Prim Care.

[17] de Barra M., Scott C.L., Scott N.W. (2018). Pharmacist services for non-hospitalised patients. Cochrane Database Syst Rev.

[18] Hayhoe B., Cespedes J.A., Foley K., Majeed A., Ruzangi J., Greenfield G. (2019). Impact of integrating pharmacists into primary care teams on health systems indicators: a systematic review. Br J Gen Pract.

[19] Hampson N., Ruane S. (2019). The value of pharmacists in general practice: perspectives of general practitioners-an exploratory interview study. Int J Clin Pharm.

[20] Hurley E., Walsh E., Foley T., Heinrich C.H., Byrne S., Dalton K. (2023). General practitioners’ perceptions of pharmacists working in general practice: a qualitative interview study. Fam Pract.

[21] Nabergoj Makovec U., Tomsic T., Kos M., Stegne Ignjatovic T., Poplas Susic A. (2023). Pharmacist-led clinical medication review service in primary care: the perspective of general practitioners. BMC Prim Care.

[22] Gutierrez Euceda B., Ferreri S.P., Armistead L.T. (2023). A descriptive analysis of primary care providers’ interest in clinical pharmacy services. Explor Res Clin Soc Pharm.

[23] Ryan K., Patel N., Lau W.M., Abu-Elmagd H., Stretch G., Pinney H. (2018). Pharmacists in general practice: a qualitative interview case study of stakeholders’ experiences in a West London GP federation. BMC Health Serv Res.

[24] Tan E.C., Stewart K., Elliott R.A., George J. (2013). Stakeholder experiences with general practice pharmacist services: a qualitative study. BMJ Open.

[25] Carayon P., Schoofs Hundt A., Karsh B.T. (2006). Work system design for patient safety: the SEIPS model. Qual Saf Health Care.

[26] Strauven G., Vanhaecht K., Anrys P., De Lepeleire J., Spinewine A., Foulon V. (2020). Development of a process-oriented quality improvement strategy for the medicines pathway in nursing homes using the SEIPS model. Res Social Adm Pharm..

[27] Carayon P., Wooldridge A., Hoonakker P., Hundt A.S., Kelly M.M. (2020). SEIPS 3.0: human-centered design of the patient journey for patient safety. Appl Ergon.

[28] Holden R.J., Carayon P. (2021). SEIPS 101 and seven simple SEIPS tools. BMJ Qual Saf.

[29] Holden R.J., Carayon P., Gurses A.P. (2013). SEIPS 2.0: a human factors framework for studying and improving the work of healthcare professionals and patients. Ergonomics.

[30] Wibeck V. (2010). Swedish.

[31] Tong A., Sainsbury P., Craig J. (2007). Consolidated criteria for reporting qualitative research (COREQ): a 32-item checklist for interviews and focus groups. Int J Qual Health Care.

[32] Donabedian A. (23 september 1988). The quality of care: how can it be assessed?. J Am Med Assoc.

[33] Russ S., Sevdalis N., Gulliford M., Jessop E. (2020). Healthcare public health: improving Health Services through Population Science [Internet].

[34] Gale N.K., Heath G., Cameron E., Rashid S., Redwood S. (2013). Using the framework method for the analysis of qualitative data in multi-disciplinary health research. BMC Med Res Methodol.

[35] Swedish Ethical Review Authority (2024). What the Act Says. https://etikprovningsmyndigheten.se/en/what-the-act-says/.

[36] Dobszai A., Lenander C., Borgström Bolmsjö B., Wickman K., Modig S. (2023). Clinical impact of medication reviews for community-dwelling patients in primary healthcare. BMC Prim Care.

[37] Hazen A., Sloeserwij V., Pouls B. (2021). Clinical pharmacists in Dutch general practice: an integrated care model to provide optimal pharmaceutical care. Int J Clin Pharm.

[38] Hazen A.C.M., Sloeserwij V.M., de Groot E. (2024). Non-dispensing pharmacists integrated into general practices as a new interprofessional model: a qualitative evaluation of general practitioners’ experiences and views. BMC Health Serv Res.

[39] Mann C., Anderson C., Boyd M. (2022). The role of clinical pharmacists in general practice in England: impact, perspectives, barriers and facilitators. Res Social Adm Pharm.

[40] Hurley E., Gleeson L.L., Byrne S., Walsh E., Foley T., Dalton K. (2022). General practitioners’ views of pharmacist services in general practice: a qualitative evidence synthesis. Fam Pract.

[41] Frederick K.D., Barenie R.E., Dill M.B., Wheeler J.S. (2022 Oct 10). Designing a pharmacist primary care certificate training program based on employer perceptions. Explor Res Clin Soc Pharm..

[42] Wei H., Horns P., Sears S.F., Huang K., Smith C.M., Wei T.L. (2022). A systematic meta-review of systematic reviews about interprofessional collaboration: facilitators, barriers, and outcomes. J Interprof Care.

[43] Le T., Toscani M., Colaizzi J. (2020). Telepharmacy: a new paradigm for our profession. J Pharm Pract.

[44] Nott S., Fleming C., Hawthorn G. (2024). A stepped wedge randomised controlled trial assessing the efficacy and patient acceptability of virtual clinical pharmacy in rural and remote Australian hospitals. BMC Health Serv Res.

[45] Malterud K., Siersma V.D., Guassora A.D. (2016). Sample size in qualitative interview studies: guided by information power. Qual Health Res.

[46] Hennink M.M., Kaiser B.N., Marconi V.C. (2017). Code saturation versus meaning saturation: how many interviews are enough?. Qual Health Res.

