# Supplementary material for: Exploring the experience of managers, employees, and pharmacists in clinical pharmacy in primary care using the SEIPS model: A focus group study
Source: Explor Res Clin Soc Pharm. 2025 Sep 14;20:100657. doi: 10.1016/j.rcsop.2025.100657 (PMC12481936; doi:10.1016/j.rcsop.2025.100657)
Supplement: Supplementary file 1 — Interview guides and interview mind maps. [file mmc1.docx]

**Appendix 1. Interview Guide**

**Introduction**

Welcome!

- My name is Lea Axelsson and I am a student in the Master of Pharmacy program at Uppsala University, where I am currently writing my thesis. I am here in collaboration with the Medicines Section in Region Kalmar County and Uppsala University, and we are grateful that you have taken the time to participate in today’s focus group.
- We have invited you to our discussion about clinical pharmacists in primary care centers, where we will talk about the experiences you have had of this collaboration over the past year.
- Since this is a focus group, we encourage conversation among you, where you discuss different themes that we will occasionally ask about. Remember that there are no wrong answers or opinions. We are interested in everyone’s perspectives, and as facilitators we are not involved in the pilot project, so please feel free to express yourselves openly.
- My job is to act as moderator, which means guiding the conversation and ensuring that we keep to the scheduled time, which is until XX:XX at the latest.
- I am joined by XX XX, who will take some notes during the discussion and may also add some questions.
- We in the study team are bound by confidentiality, but we also ask you to respect what is discussed here today and keep it within the group.
- You have previously received information about how the material will be used, including in a thesis, possibly in an internal report for the Region, and potentially in a publication in a scientific journal. Does anyone have any questions about this?
- (Collect the signed informed consent for participation.)

**Ice-breaker**

Round of introductions so everyone around the table gets to know each other: name, profession, workplace, and what you had for breakfast.

**Start recording**

Let us begin with the questions for this focus group. From now on, we will not go around the table one by one; instead, anyone may speak whenever they wish.


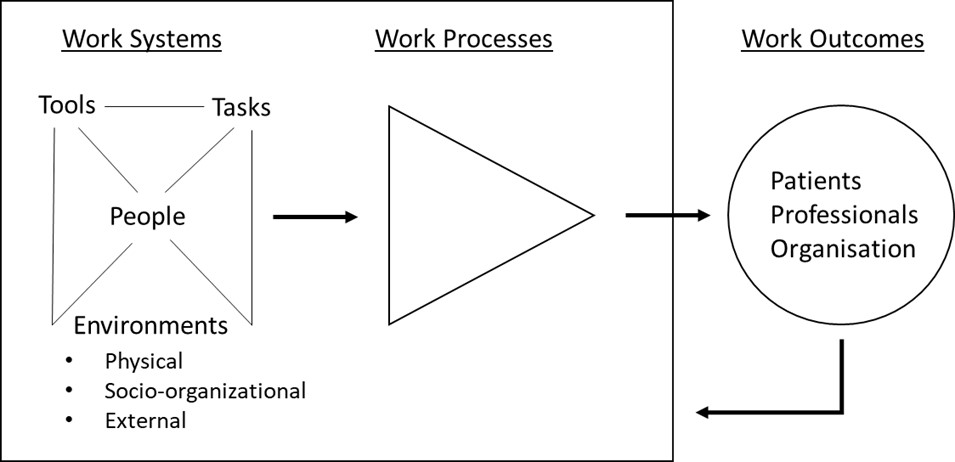


|  | | | |
| --- | --- | --- | --- |
| **Guide and Mindmap for Focus Group with Pharmacists** | | | |
| **Main theme** | | **Core question** | **Supporting questions (not all need to be asked)** |
| **Work system** | **People**  Individuals or groups of people. Their physical, cognitive (knowledge), and psychosocial (motivation) characteristics.  *Pharmacists* | How do you contribute with your pharmaceutical competence to the competence that already existed at the primary care center?  *By competence we mean pharmaceutical knowledge, skills, experience, and behavior.* | - Which competencies have you brought into new areas of work? - Are there additional competencies you would have wished to have in order to work in primary care? Have you gained or adapted competencies from other workplaces? - What motivated you to participate in the project with a pharmacist at the primary care center? - Expectations of the collaboration in healthcare? |
|  | **Environment**  Settings for activity within the analysis unit (pharmacists in primary care) or the surrounding external context, and the characteristics and influences of these environments.  **Physical**  *Physical layout, location, lighting, sound, temperature.*  **Organizational**  Descriptions of an organizational unit (hospital, health center, home), such as structure, procedures, roles, responsibilities, relationships, and organizational culture. Conditions and prerequisites for being able to work at the primary care center. For example: social support from managers/colleagues, communication → how work is directed/how decisions are made, activities.  **External**  *The external environment is that which influences the unit from outside, for example regulatory, legal, economic, political, cultural, or societal contexts.* | How has it worked in the organizational environment of primary care to establish collaboration at the primary care center?  By organizational we mean the conditions and prerequisites provided to work at a primary care center. Examples: how work is directed, how decisions are made, social support from managers and colleagues, communication. | - How clear is the pharmacist’s role at the primary care center to the employees? How could it be developed? - Improvement opportunities for the new implementation of a pharmacist in the primary care organization? - Have you as a pharmacist been established in the physical environment at the primary care center? |
|  | ****Task**** *Specific actions in a process, and the characteristics of those actions.* | Can you describe what you have worked on when based at the primary care center? For example, specific tasks. | - How often have you been at the primary care center? - In what areas do you think you have contributed the most? - Who has been most involved in deciding your tasks at the primary care center? - In addition to what has already been done, what further tasks do you think a pharmacist could work with in the future at the primary care center? Considering: - Patient care, Colleagues, The organization |
|  | ****Tools**** Objects used to perform a job. For example, information technology or medical equipment (usability, accessibility, automation, functionality). | Have you experienced any obstacles in carrying out your tasks because, for example, you did not have access to the right systems? | - For example, computers, access to medical records. - Any tool/system that has been missing in your work? |
| ****Process**** Work processes are how work is performed and how it flows. Work processes are physical, cognitive, social, behavioral, or a combination. They may be carried out by healthcare professionals, patients and families, or in collaboration between professionals and non-professionals. How other work processes influence this process. | | Can you tell us a bit about how you have collaborated with the staff at the primary care center? How has the collaboration looked? Describe a day at the primary care center. | - What has it been like as a pharmacist to enter the primary care center? - What has worked well and what has not worked well? - Which contact routes have been used? (telephone, chat, office) - Easy to reach each other? Who contacts whom? - How could the collaboration have been developed further? |
| **Outcomes**  Outcomes are the result of the work system and work processes. These may be desirable or undesirable. They affect professionals, patients/families, or the organization.   - **Patient/family** - **Healthcare staff/colleagues** - **Organization** | | What is your perception of how you have contributed to patients’ care and to their ability to manage and use medicines themselves?  And what could be contributed in the future beyond that? | - How do you think you as a pharmacist have contributed to your colleagues at the primary care center? - And to their way of working with medicine-related issues and problems? - Thinking about the entire primary care organization in Region Kalmar County: - Where do you think there may be a beneficial role for the pharmacist profession? - Do you think pharmacists’ work with medication-related issues in primary care should be organized in a different way? - Regarding the ongoing documentation of the effects of pharmacists at the primary care center - Thoughts on the follow-up and whether it should be done in a different way? - Which data do you consider important to demonstrate the effect of pharmacists in primary care? Which outcome measures are important? - Considering the systems used today, is it possible to document the effect needed, or would a new function need to be added? - Is there a need to expand the number of pharmacists in primary care? |


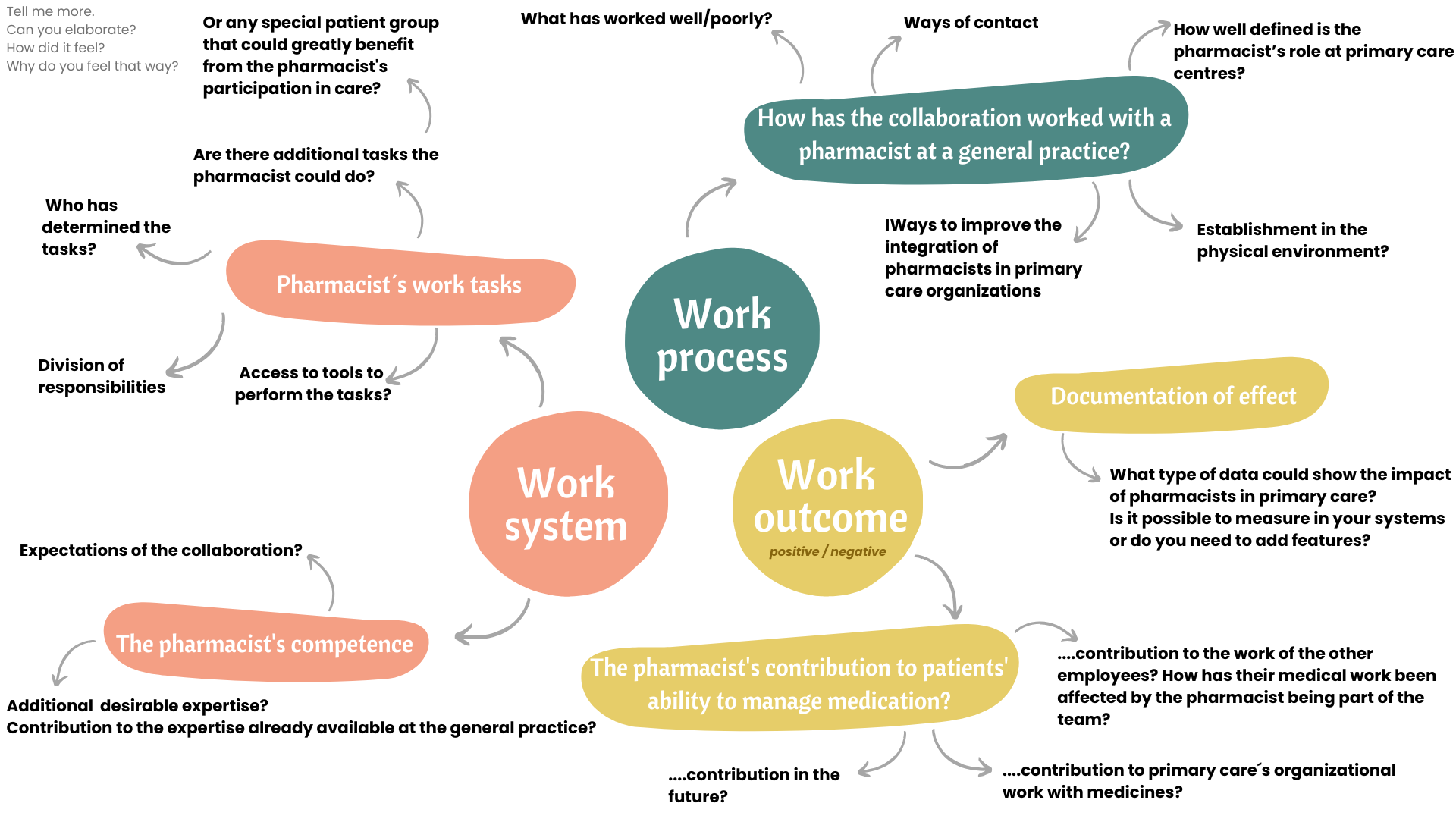


| **Guide and Mindmap for Focus Group with Employees** | | | |
| --- | --- | --- | --- |
| **Huvudtema** | | **Kärnfråga** | **Stödfrågor (alla behöver inte ställas)** |
| **Work system** | **People**  Individuals or groups of people. Their physical, cognitive (knowledge), and psychosocial (motivation) characteristics.  *Pharmacists* | Can you describe what competence the pharmacist has contributed with in addition to what already exists at the primary care center?  By competence we mean pharmaceutical knowledge, skills, experience, and behavior. | - Are there additional competencies you would wish the pharmacist had? - What were your expectations before the collaboration with pharmacists in primary care? |
|  | **Environment**  Settings for activity within the analysis unit (pharmacists in primary care) or the surrounding external context, and the characteristics and influences of these environments.  **Physical**  *Physical layout, location, lighting, sound, temperature.*  **Organizational**  Descriptions of an organizational unit (hospital, health center, home), such as structure, procedures, roles, responsibilities, relationships, and organizational culture. Conditions and prerequisites for being able to work at the primary care center. For example: social support from managers/colleagues, communication → how work is directed/how decisions are made, activities.  **External**  *The external environment is that which influences the unit from outside, for example regulatory, legal, economic, political, cultural, or societal contexts.* | How has it worked in the organizational environment of primary care to establish collaboration at the primary care center?  By organizational we mean the conditions and prerequisites provided to work at a primary care center. Examples: how work is directed, how decisions are made, social support from managers and colleagues, communication. | - How clear is the pharmacist’s role at the primary care center to you and the employees? How could it be developed? - Improvement opportunities when newly implementing a pharmacist in the primary care organization? - Have the pharmacist physical been established at the work place? |
|  | ****Task**** *Specific actions in a process, and the characteristics of those actions.* | What tasks has the pharmacist worked on at your primary care center? | - How often was the pharmacist at your PCC? - What did you not expect to receive help with from a pharmacist? - Who has been most involved in deciding which tasks the pharmacist would work on at your primary care center? And who should that be? - In addition to what has already been done, what further tasks do you think a pharmacist could work with in the future at the primary care center? Considering: - Patient care - Colleagues - The organization |
|  | ****Tools**** Objects used to perform a job. For example, information technology or medical equipment (usability, accessibility, automation, functionality). | Have you experienced any obstacles for the pharmacist in carrying out tasks, for example because access to systems did not work? | - For example, computers, access to medical records. - Any tool/system that has been missing in the work? |
| ****Process**** Work processes are how work is performed and how it flows. Work processes are physical, cognitive, social, behavioral, or a combination. They may be carried out by healthcare professionals, patients and families, or in collaboration between professionals and non-professionals. How other work processes influence this process. | | Can you tell us a bit about how the collaboration with the pharmacist at the primary care center has looked? I am thinking about your one-to-one collaboration. Tell me about a day at the health care center. | - What has worked well and what has not worked well? - Which contact routes have been used? (telephone, chat, office) - Easy to reach each other? Who contacts whom? - How could the collaboration have been developed further? - How has the work with medication issues/routines/work processes at the primary care center been influenced after a pharmacist was employed? - Do you perceive that the pharmacist has found a natural role at the primary care center? |
| **Outcomes**  Outcomes are the result of the work system and work processes. These may be desirable or undesirable. They affect professionals, patients/families, or the organization.   - **Patient/family** - **Healthcare staff/colleagues** - **Organization** | | What is your perception of how the pharmacist has contributed to patients’ care and to their ability to manage and use medicines themselves?  And what could be contributed in the future beyond that? | - How do you think the pharmacist has contributed to your colleagues at the primary care center? - And to the primary care center’s way of working with medicine-related issues and problems? - Thinking about the entire primary care organization in Region Kalmar County: - Where do you think there may be a beneficial role for the pharmacist profession? - Do you think pharmacists’ work with medication-related issues in primary care should be organized in a different way?   Regarding the ongoing documentation of the effects of pharmacists at the primary care center   - Thoughts on the follow-up and whether it should be done in a different way? - Which data do you consider important to demonstrate the effect of pharmacists in primary care? Which outcome measures are important? - Considering the systems used today, is it possible to document the effect needed, or would a new function need to be added?   Is there a need to expand the number of pharmacists in primary care? |


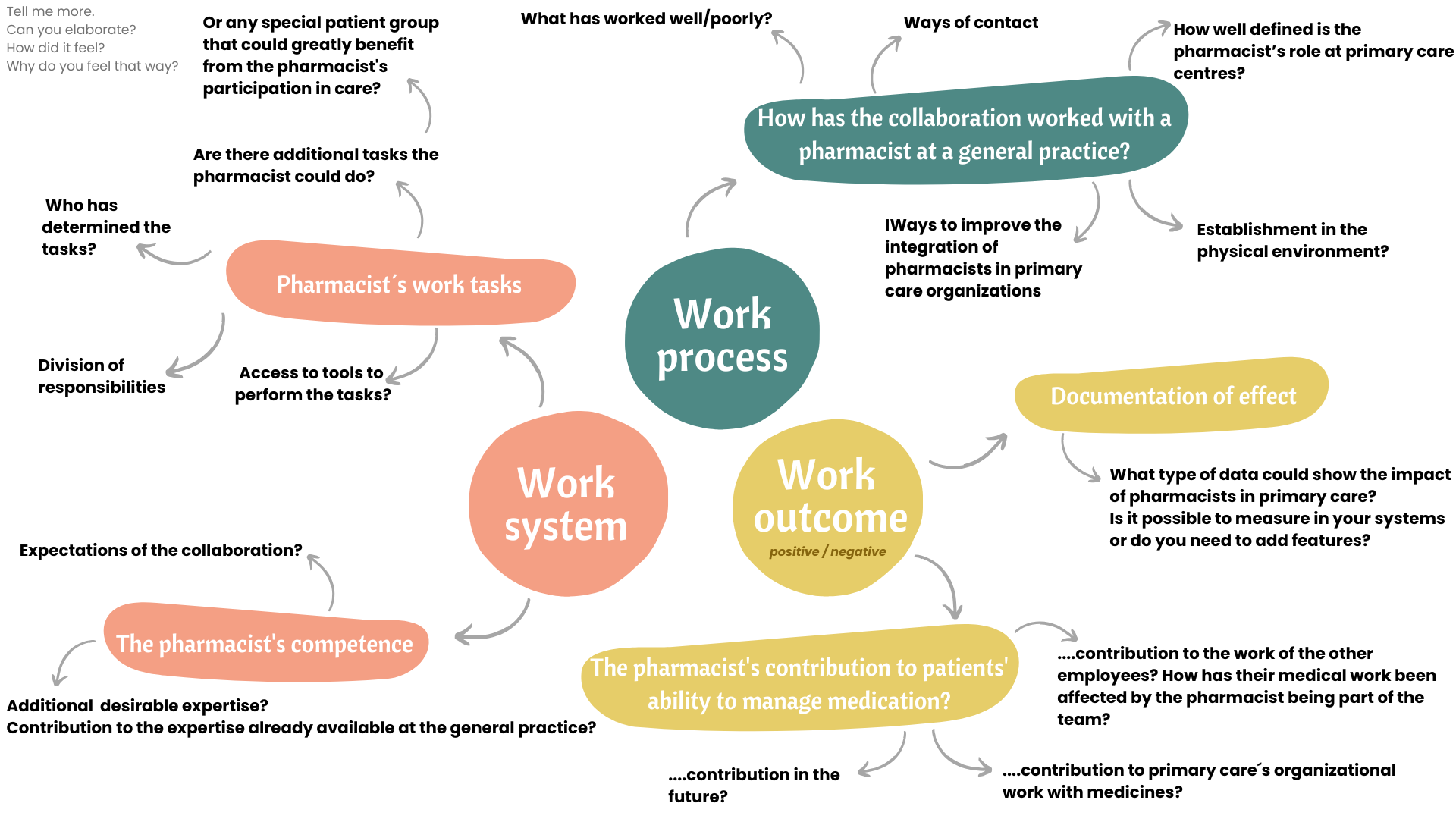


| **Guide and mindmap for focus group with managers** | | | |
| --- | --- | --- | --- |
| **Main theme** | | **Core question** | **Supporting questions (not all need to be asked)** |
| **Work system** | **People**  Individuals or groups of people. Their physical, cognitive (knowledge), and psychosocial (motivation) characteristics.  *Pharmacists* | Can you describe what competence the pharmacist has contributed with in addition to what already exists at the primary care center?  By competence we mean pharmaceutical knowledge, skills, experience, and behavior. | - Are there additional competencies you would wish the pharmacist had? - What were your expectations before the collaboration with pharmacists in primary care? |
|  | **Environment**  Settings for activity within the analysis unit (pharmacists in primary care) or the surrounding external context, and the characteristics and influences of these environments.  **Physical**  *Physical layout, location, lighting, sound, temperature.*  **Organizational**  Descriptions of an organizational unit (hospital, health center, home), such as structure, procedures, roles, responsibilities, relationships, and organizational culture. Conditions and prerequisites for being able to work at the primary care center. For example: social support from managers/colleagues, communication → how work is directed/how decisions are made, activities.  **External**  *The external environment is that which influences the unit from outside, for example regulatory, legal, economic, political, cultural, or societal contexts.* | How has it worked in the organizational environment of primary care to establish collaboration at the primary care center?  By organizational we mean the conditions and prerequisites provided to work at a primary care center. Examples: how work is directed, how decisions are made, social support from managers and colleagues, communication. | - How clear is the pharmacist’s role at the primary care center to you and the employees? How could it be developed? - Improvement opportunities when newly implementing a pharmacist in the primary care organization? |
|  | ****Task**** *Specific actions in a process, and the characteristics of those actions.* | What tasks has the pharmacist worked on at your primary care center? | - What did you not expect to receive help with from a pharmacist? - Who has been most involved in deciding which tasks the pharmacist would work on at your primary care center? - In addition to what has already been done, what further tasks do you think a pharmacist could work with in the future at the primary care center? Considering: - Patient care - Colleagues - The organization |
|  | ****Tools**** Objects used to perform a job. For example, information technology or medical equipment (usability, accessibility, automation, functionality). | Have you experienced any obstacles for the pharmacist in carrying out tasks, for example because access to systems did not work? | - For example, computers, access to medical records. - Any tool/system that has been missing in the work? |
| ****Process**** Work processes are how work is performed and how it flows. Work processes are physical, cognitive, social, behavioral, or a combination. They may be carried out by healthcare professionals, patients and families, or in collaboration between professionals and non-professionals. How other work processes influence this process. | | Can you tell us a bit about how the collaboration with the pharmacist at the primary care center has looked? I am thinking about your one-to-one collaboration. | - What has worked well and what has not worked well? - Which contact routes have been used? (telephone, chat, office) - Easy to reach each other? Who contacts whom? - How could the collaboration have been developed further? - How has the work with medication issues/routines/work processes at the primary care center been influenced after a pharmacist was employed? - Do you perceive that the pharmacist has found a natural role at the primary care center? |
| **Outcomes**  Outcomes are the result of the work system and work processes. These may be desirable or undesirable. They affect professionals, patients/families, or the organization.   - **Patient/family** - **Healthcare staff/colleagues** - **Organization** | | What is your perception of how the pharmacist has contributed to patients’ care and to their ability to manage and use medicines themselves?  And what could be contributed in the future beyond that? | - How do you think the pharmacist has contributed to your colleagues at the primary care center? - And to the primary care center’s way of working with medicine-related issues and problems? - Thinking about the entire primary care organization in Region Kalmar County: - Where do you think there may be a beneficial role for the pharmacist profession? - Do you think pharmacists’ work with medication-related issues in primary care should be organized in a different way?   Regarding the ongoing documentation of the effects of pharmacists at the primary care center   - Thoughts on the follow-up and whether it should be done in a different way? - Which data do you consider important to demonstrate the effect of pharmacists in primary care? Which outcome measures are important? - Considering the systems used today, is it possible to document the effect needed, or would a new function need to be added? - Is there a need to expand the number of pharmacists in primary care? |


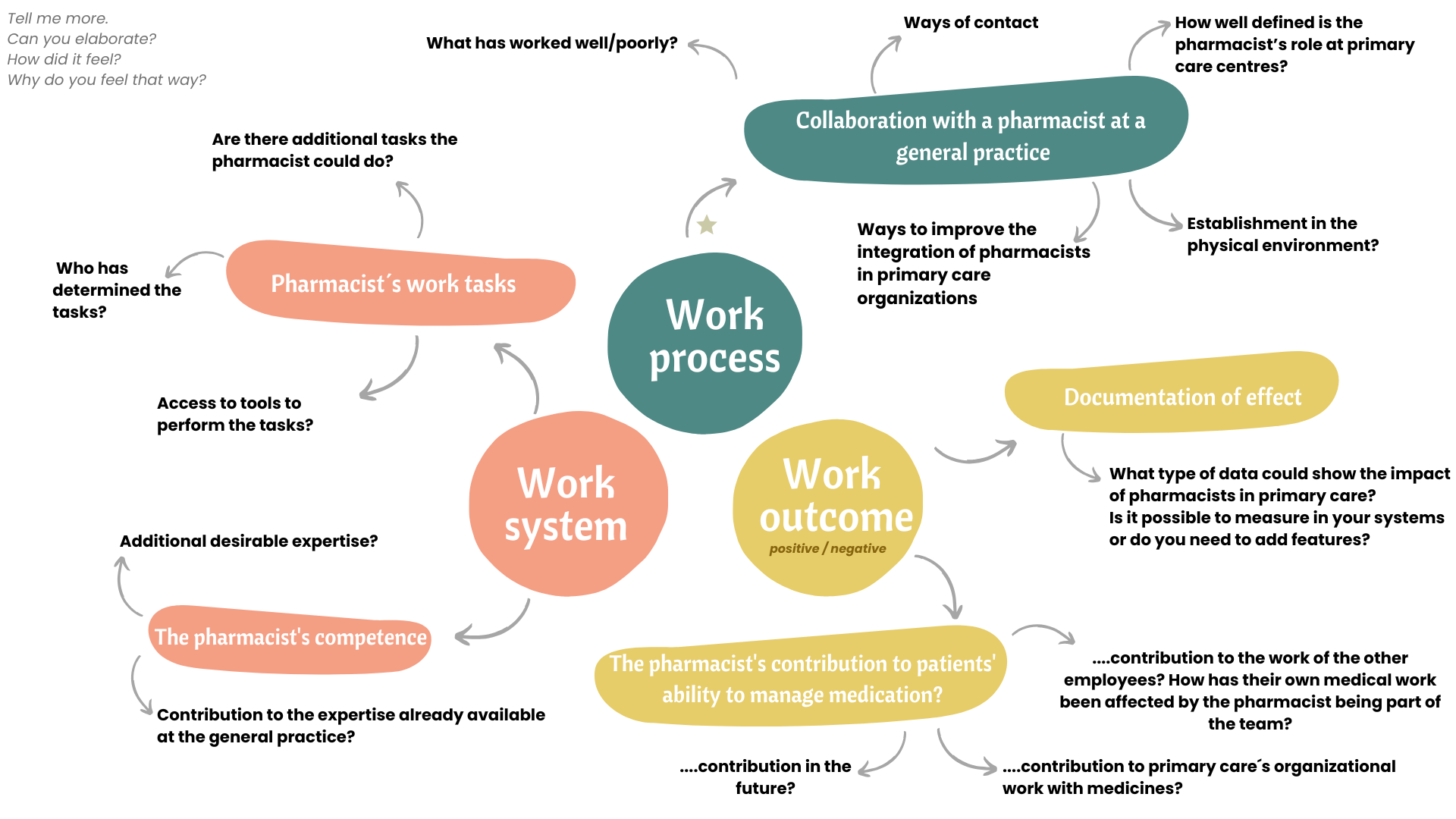


## **Winding down**

- Is there anything anyone would like to add beyond what we have discussed today?
- Ask the co-moderator if they have any further questions for the group, and then provide a summary of what has been brought up during the discussion.

## **Conclusion**

Thank you very much for participating in this focus group study.

You have previously received information about how the material will be used, but going forward the same applies. If you have any questions, please feel free to contact me or one of my supervisors.

Are there any final questions?

**Stop recording**
